# The optimal surgery timing after stenting in colorectal cancer patients with malignant obstruction: additionally compared with emergency surgery

**DOI:** 10.1186/s12957-023-03130-6

**Published:** 2023-08-23

**Authors:** Ji Eun Na, Eun Ran Kim, Ji Eun Kim, Sung Noh Hong, Young Ho Kim, Dong Kyung Chang

**Affiliations:** 1 Department of Medicine, Samsung Medical Center, Sungkyunkwan University School of Medicine, 81 Irwon-ro, Gangnam-gu, Seoul, 06351 Korea; 2 Department of Medicine, Inje University Haeundae Paik Hospital, Busan, Korea

**Keywords:** Colorectal cancer, Malignant obstruction, Self-expandable metal stent, Optimal timing of surgery

## Abstract

**Background:**

This study aimed to determine short-term and long-term outcomes according to time intervals after stenting and compared them with those of emergency surgery (ES) in colorectal cancer (CRC) with malignant obstruction.

**Methods:**

CRC with malignant obstructions was reviewed retrospectively between January 2008 and July 2018. Of a total of 539 patients who visited the emergency room and underwent ES, 133 were enrolled in the ES group. Of a total of 567 patients who initially received stenting and subsequently underwent elective surgery, 220 were enrolled in the SEMS group. The interval between SEMS placement and elective surgery was classified as < 11 days, 11–17 days, and > 17 days.

**Results:**

For those who received SEMS (*n* = 220), those with a time interval of 11–17 days (*n* = 97) had fewer hospital days than those with a time interval of < 11 days (*n* = 68) (8 days vs. 15 days) and less stoma formation than those with a time interval of > 17 days (*n* = 55) (1.0% vs. 14.6%). Multivariable analysis revealed a decreased risk of death for the group with a time interval of 11–17 days (20.6%) compared to the ES group (31.6%) (hazard ratio: 0.48; 95% confidence interval: 0.24–0.97). Disease-free survival was comparable between the SEMS and ES groups regardless of the time interval (log-rank *p* = 0.52).

**Conclusions:**

The time interval of 11–17 days after stenting to elective surgery appeared to be associated with the most favorable outcomes.

**Supplementary Information:**

The online version contains supplementary material available at 10.1186/s12957-023-03130-6.

## Introduction

Self-expandable metal stents (SEMS) serve as a bridge to surgery in colorectal cancer (CRC) patients with malignant obstruction accompanied by symptoms and suggestive radiologic findings, in contrast to emergency surgery (ES) [1]. SEMS offers advantages over ES, including lower rates of postoperative complications [2–6] and stoma formation [3–8]. SEMS also allows for the endoscopic evaluation of synchronous malignant or premalignant lesions before elective surgery [9, 10] and enables bowel preparation, which can reduce the risk of surgical site infection and anastomosis leakage [11–13]. However, an ongoing debate about its oncological safety remains. Although some meta-analyses showed no significant difference in tumor recurrence between SEMS and ES [7, 14–17], other meta-analyses reported a higher recurrence risk in SEMS than in ES [2, 3]. Contributing factors to such differences in results included the time interval from stenting to elective surgery. Therefore, it is important to determine the ideal interval from stenting to elective surgery to balance the benefits and oncologic safety concerns of SEMS.

The optimal time interval between stenting and elective surgery remains uncertain. The updated European Society of Gastrointestinal Endoscopy (ESGE) guidelines recommend an interval of 2 weeks rather than the previous range of 5–10 days [1]. In a subsequent nationwide study, the time interval of 11–17 days showed no difference in postoperative complications or long-term outcomes compared to a time interval of 5–10 days, whereas the time interval of 5–10 days showed a lower proportion of laparoscopic resections compared to a time interval of > 17 days [18]. These results support the revised guidelines. Inconsistently, another study revealed that the short-term and long-term outcomes were favorable in the group with a time interval of < 8 days compared to 8–14 days or > 14 days [19]. Hence, additional evidence is needed to determine the optimal timing of surgery after stenting. Studies to date have only focused on comparing time intervals from stenting to elective surgery. It is necessary to evaluate whether there is a difference between time intervals and ES.

Therefore, this study aimed to assess whether there were differences in short-term and long-term outcomes according to time intervals after stenting. Additionally, the outcomes of SEMS were compared to those of ES.

### Subjects and methods

#### Patients

This retrospective single-center study examined patients who underwent radical surgery for CRC with malignant bowel obstruction between January 2005 and July 2018. In the ES group, patients who visited the emergency room and subsequently underwent surgery were screened. The exclusion criteria of the ES group were concurrent complications, such as bleeding or perforation, distant metastasis, and missing values for key variables. Patients who underwent stenting for malignant bowel obstruction and subsequent surgery were screened for the SEMS group during the same period. The patients indicated for stenting had obstructive symptoms, verified obstruction that could not be passed by endoscopy, and suggestive imaging findings. Cases with distant metastasis, missing values for major variables, and no subsequent surgery after stenting were excluded.

The Institutional Review Board (IRB) of Samsung Medical Center (Korea) approved this study (IRB File Number: 2020–06-090–004). The requirement to acquire informed consent was waived by the IRB due to the retrospective nature of the study.

#### Perioperative management and surveillance

The initial workup for clinical staging consisted of carcinoembryonic antigen (CEA) testing, colonoscopy if feasible, chest computed tomography (CT), abdominal and pelvic CT, and rectum magnetic resonance imaging (MRI) exclusively for rectal cancer.

SEMS placement was indicated in patients with obstructive symptoms, severe obstruction proved by endoscopy, and ileal or upstream dilatation seen on radiological images. All procedures were conducted in a dedicated room under fluoroscopic guidance. After assuming stenosis length using endoscopy and fluoroscopy by infusing radiocontrast dye, SEMS were deployed at the site of the obstruction. Immediately after SEMS placement, X-ray was used to evaluate the occurrence of perforation.

The surgeon planned colorectal surgery with lymph node dissection according to the tumor location and clinical stage. Neoadjuvant therapy or adjuvant therapy was recommended based on current guidelines [20, 21].

Surveillance after surgery included basic blood tests (including CEA), chest CT, and abdominal/pelvic CT every 6 months for 5 years. Endoscopic surveillance was performed 6 months to 1 year after surgery and then every 2 years or adjusted based on reports of endoscopy during the 5 years.

#### Assessment of outcomes and data collection

The primary outcome was the comparison of short-term outcomes among time interval groups from stenting to elective surgery and between the SEMS and ES groups. The surgeon determined the timing of elective surgery based on various clinical factors, including preoperative risk evaluation for comorbidities, the patient’s overall recovery, compliance/complications after stenting, and the surgeon’s schedule. Time intervals were classified into < 11 days, 11–17 days, and > 17 days. Short-term outcomes were assessed by comparing surgical results, such as by the laparoscopic approach, stoma formation, operation time, estimated blood loss (EBL), harvested lymph nodes (LNs), positive LNs, major adverse events (Dindo-Clavien classification ≥ grade III) within 90 days and the entire follow-up period after surgery, hospital stays after admission for surgery, and adjuvant chemotherapy.

The secondary outcome was a comparison of long-term outcomes and identifying the risk factors for mortality and recurrence. Long-term outcomes were defined as overall survival (OS) and disease-free survival (DFS), which were compared among the time intervals (< 11 days, 11 – 17 days, and > 17 days) and with ES. OS was defined as survival from the index date of surgery to death. Survival status was obtained from the Statistics Korea database. DFS was defined as survival from the index surgery date to the date of the first relapse or the last follow-up, whichever occurred first. Colorectal cancer relapse was diagnosed as local or distant recurrence by follow-up endoscopy with biopsy or abdominal and pelvic CT. Data on survival and recurrence were collected until July 2021.

The following variables were retrospectively reviewed in the electronic medical records: age, sex, body mass index (BMI), American Society of Anesthesiologists (ASA) classification for the assessment of patient comorbidities, preoperative CEA level, neoadjuvant and adjuvant chemotherapy, location (right, left, and rectum), pathologic tumor size (cm), tumor histology, TNM staging, lymphovascular invasion, perineural invasion, microsatellite instability (MSI), and the surgical outcomes mentioned above.

#### Statistical analysis

Baseline characteristics and short-term outcomes were compared using a variety of statistical tests. Continuous variables were analyzed using the Student’s *t* test, Wilcoxon rank sum test, and Tukey’s test after rank transformation. Categorical variables were analyzed using the chi-squared and Fisher’s exact test with the Bonferroni method. Kaplan–Meier analysis was used to compare OS and DFS. Cox proportional hazards regression analysis was utilized to find potential risk factors for long-term outcomes. Variables that showed a *P* value of less than 0.1 in the univariable analysis were included in the multivariable analysis. Time interval subgroups of the SEMS and ES groups, which were of interest, were also included in the multivariable analysis regardless of their association in univariable analysis. *P* values and 95% confidence intervals (CI) were corrected using Bonferroni’s method to account for multiple comparisons. Multicollinearity was checked using the variance inflation factor. There were no variables with a variance inflation factor > 4. *P* values of < 0.05 were considered statistically significant. All statistical analyses were performed using SAS version 9.4 (SAS Institute, Cary, NC, USA) and R 3.5.1 (The R Foundation for Statistical Computing, Vienna, Austria).

## Results

### Baseline characteristics of the study population

**Fig. 1:**
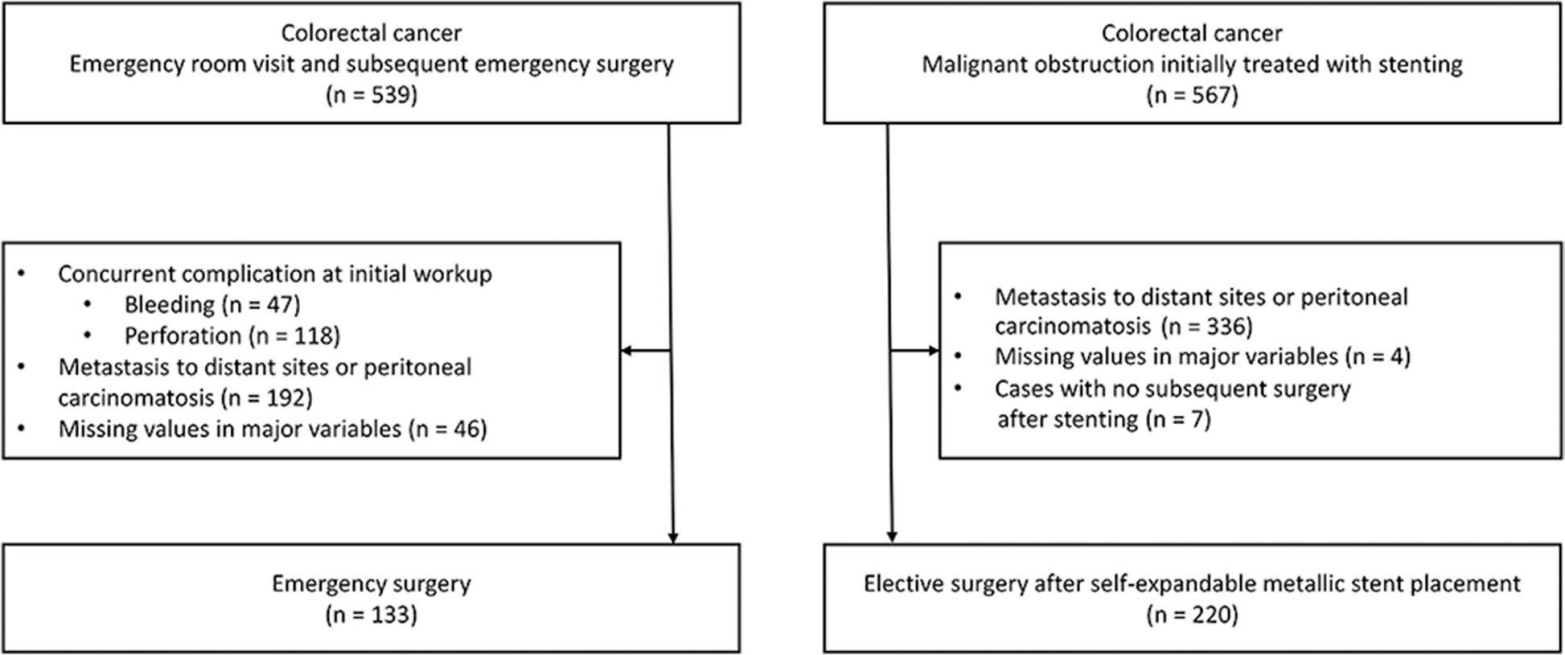
Flow chart showing the selection of patients for the study

A total of 353 patients were enrolled based on our inclusion and exclusion criteria, with 133 (37.7%) patients in the ES group and 220 (62.3%) patients in the SEMS group out of 539 and 567 screened patients, respectively (Fig. 1). Patients in the SEMS group had older age, a higher proportion of left side locations, larger pathologic sizes, and a higher proportion of peri-neural invasion but a lower proportion of high MSI and undifferentiated histology than the ES group. In the comparison between the ES group and time interval groups of SEMS, there were no statistically significant changes compared to the comparison between the ES and SEMS groups, except for the proportions of venous invasion and high MSI (Table 1).

**Table 1:** Clinic-pathologic characteristics among emergency surgery, self-expandable metal stents, and stratified time intervals of the stent

	ES (*n* = 133)	SEMS (*n* = 220)	*P*^a^	< 11 (*n* = 68)	*P*^b^	11–17 (*n* = 97)	*P*^c^	> 17 (*n* = 55)	*P*^d^	P^*^	P**	P***
Age, years†	60 (51, 71)	67 (57, 76)	< 0.001	67 (55, 76)	0.025	65 (57, 74)	0.003	69 (57, 78)	< 0.001	0.10	0.48	0.15
Sex			0.36		0.81		0.49		0.23	0.99	0.99	0.38
Male	72 (54.1)	130 (59.1)		38 (55.9)		57 (58.8)		35 (63.6)				
Female	61 (45.9)	90 (40.9)		30 (44.1)		40 (41.2)		20 (36.4)				
BMI	23 (21, 25)	22 (20, 25)	0.08	22 (20, 24)	0.16	22 (21, 24)	0.19	22 (19, 25)	0.17	0.95	0.95	0.99
ASA III	8 (6.0)	22 (10.0)	0.19	4 (5.9)	0.99	7 (7.2)	0.72	11 (20.0)	0.004	0.99	0.07	0.017
Preoperative CEA†	2.7 (1.6, 6.9)	2.8 (1.4, 5.4)	0.54	2.1 (1.2, 4.9)	0.43	3.4 (1.7, 7.3)	0.60	2.3 (1.2, 4.4)	0.55	0.034	0.09	0.63
Neoadjuvant chemotherapy	0 (0.0)	6 (2.7)	0.09	0 (0.0)	–	0 (0.0)	–	6 (10.9)	0.001	0.99	0.004	0.007
Location			< 0.001		< 0.001		< 0.001		< 0.001	0.40	0.88	0.12
Right	71 (53.4)	25 (11.4)		11 (16.2)		8 (8.2)		6 (10.9)				
Left	46 (34.6)	175 (79.6)		54 (79.4)		80 (82.5)		41 (74.6)				
Rectum	16 (12.0)	20 (9.1)		3 (4.4)		9 (9.3)		8 (14.5)				
Pathologic size, cm†	5.5 (4.5, 7.0)	7.0 (6.0, 8.0)	< 0.001	7.0 (6.0, 7.8)	0.003	7.0 (6.0, 8.0)	< 0.001	7.0 (6.0, 8.0)	0.004	0.33	0.58	0.71
Undifferentiated histology	22 (16.5)	10 (4.6)	< 0.001	5 (7.4)	0.07	3 (3.1)	0.001	2 (3.6)	0.016	0.55	0.99	0.46
Lymphatic invasion, present	47 (35.9)	86 (39.1)	0.55	23 (33.8)	0.77	38 (39.2)	0.61	25 (45.5)	0.22	0.99	0.99	0.19
Venous invasion, present	23 (20.9)	35 (15.9)	0.26	4 (5.9)	0.007	22 (22.7)	0.76	9 (16.4)	0.49	0.008	0.81	0.06
Peri-neural invasion, present	37 (29.8)	133 (60.5)	< 0.001	36 (52.9)	0.002	65 (67.0)	< 0.001	32 (58.2)	< 0.001	0.15	0.59	0.56
MSI, high	14 (12.5)	10 (4.7)	0.011	3 (4.6)	0.08	2 (2.1)	0.005	5 (9.6)	0.59	0.80	0.19	0.30
T stage			< 0.001		0.003		0.015		< 0.001	0.31	0.07	0.50
T2	2 (1.5)	3 (1.4)		2 (2.9)		0 (0.0)		1 (1.8)				
T3	65 (48.9)	156 (70.9)		48 (70.6)		64 (66.0)		44 (80.0)				
T4	66 (49.6)	61 (27.7)		18 (26.5)		33 (34.0)		10 (18.2)				
N stage			0.53		0.52		0.80		0.66	0.99	0.99	0.85
N0	56 (42.1)	106 (48.2)		34 (50.0)		45 (46.4)		27 (49.1)				
N1	53 (39.9)	77 (35.0)		22 (32.4)		35 (36.1)		20 (36.4)				
N2	24 (18.0)	37 (16.8)		12 (17.6)		17 (17.5)		8 (14.5)				
Pathologic stage			0.27		0.29		0.52		0.38	0.99	0.99	0.92
I–II	56 (42.1)	106 (48.2)		34 (50.0)		45 (46.4)		27 (49.1)				
III	77 (57.9)	114 (51.8)		34 (50.0)		52 (53.6)		28 (50.9)				
Follow-up duration, month^†^	64 (22, 95)	36 (14, 59)	< 0.001	46 (13, 63)	< 0.001	37 (15, 57)	< 0.001	30 (13, 44)	< 0.001	0.51	0.53	0.010

Values are expressed as *n* (%) unless otherwise specified

*ASA* American Society of Anesthesiologists, *BMI* body mass index, *CEA* Carcinoembryonic antigen, *ES* emergency surgery, *MSI* microsatellite instability, *SEMS* self-expendable metal stent

^†^Value is median (IQR, interquartile range)

*P* value calculated using Student’s *t* test and Wilcoxon rank sum test for continuous variables or chi-square test and Fisher’s exact test for categorical variables

^a,b,c,d^*P* value was a comparison of emergency surgery and the time interval groups with < 11 days, 11–17 days, and > 17 days, respectively

^*, **, ***^P value was a comparison of the time interval groups with < 11 days and 11–17 days, 11–17 days, and > 17 days, and < 11 days and > 17 days, respectively. Those were compared using Tukey’s test after rank transformation and Fisher’s exact test with the Bonferroni method

Among the 220 patients who underwent stenting as a bridge to surgery, 68 (30.9%) patients were in the group with a time interval of < 11 days, 97 (44.1%) patients were in the group with a time interval of 11–17 days, and 55 (25.0%) patients were in the group with a time interval of > 17 days. Four (1.8%) patients experienced gross or micro-perforation after stenting and underwent emergency surgery. They were included in the group with a time interval of < 11 days. The group with a time interval of 11–17 days had a higher proportion of venous invasion than that of < 11 days. Other variables were comparable between time intervals of < 11 days and 11–17 days. Groups with time intervals of 11–17 days and > 17 days also showed similar characteristics except for neoadjuvant chemotherapy. All six patients who received neoadjuvant chemotherapy belonged to the group with a time interval of > 17 days. The group with a time interval of > 17 days had a higher proportion of ASA III compared to those with < 11 days.

### Short-term outcomes

The SEMS group showed a significantly higher proportion of laparoscopic approaches, lower EBL, and fewer hospital days than the ES group. In comparison, the ES group had a higher proportion of stoma formation, longer operation time, and more occurrence of postoperative complications within 90 days or the entire follow-up period than the SEMS group, although these differences were not statistically significant between the two groups (Table 2).

**Table 2:** Comparison of short-term outcomes among emergency surgery, self-expandable metal stents, and stratified time intervals of stents

	ES (*n* = 133)	SEMS (*n* = 220)	*P*^a^	< 11 (*n* = 68)	11–17 (*n* = 97)	> 17 (*n* = 55)	P^*^	P^**^	P^***^
Laparoscopic approach	5 (3.8)	82 (32.3)	< 0.001	16 (23.5)	39 (40.2)	27 (49.1)	0.06	0.62	0.003
Stoma formation	10 (7.5)	12 (5.5)	0.44	3 (4.4)	1 (1.0)	8 (14.6)	0.61	0.003	0.06
Operation time, min	152 (126, 200)	144 (115, 191)	0.19	144 (100, 250)	142 (113, 180)	149 (117, 221)	0.98	0.66	0.21
Estimated blood loss, ml	150 (100, 250)	100 (50, 150)	< 0.001	100 (50, 200)	100 (50, 100)	100 (50, 200)	0.20	0.49	0.98
Harvested LN, number	24 (16, 31)	26 (20, 34)	0.039	26 (19, 34)	26 (22, 33)	24 (19, 34)	0.90	0.68	0.53
Positive LN, number	1 (0, 2)	1 (0, 2)	0.30	1 (0, 3)	1 (0, 2)	1 (0, 2)	0.96	0.93	0.24
Surgery-related complication, < 90 days	12 (9.0)	9 (4.1)	0.06	4 (5.9)	3 (3.1)	2 (3.6)	0.90	0.99	0.69
Major adverse event (≥ CD G3)	18 (13.5)	18 (8.2)	0.11	4 (5.9)	8 (8.3)	6 (10.9)	0.99	0.99	0.34
Leakage	0	3		0	2	1			
Dehiscence	3	2		2	0	0			
Wound infection	3	0		0	0	0			
Abscess	2	1		0	1	0			
Fistula	1	1		0	1	0			
Adhesive ileus	2	3		0	1	2			
Ureter stricture	5	7		2	2	3			
Stenosis	1	0		0	0	0			
Hernia	0	1		0	1	0			
Ischemia	1	0		0	0	0			
Hospital days^†^	12 (10, 16)	9 (7, 15)	< 0.001	15 (10, 18)	8 (7, 10)	10 (8, 13)	< 0.001	0.10	0.34
Adjuvant chemotherapy	95 (71.4)	128 (58.2)	0.012	47 (69.1)	57 (58.8)	24 (43.6)	0.39	0.18	0.004

Values are expressed as *n* (%) unless otherwise specified

*CD Grade* Clavien-Dindo classification, *ES* emergency surgery, *LN* lymph node, *SEMS* self-expendable metal stent

^†^Value is median (IQR, interquartile range)

^a^*P* value calculated using Student’s *t* test and Wilcoxon rank sum test for continuous variables or chi-square test and Fisher’s exact test for categorical variables

^*, **, ***^P value was a comparison of the time interval groups with < 11 days and 11–17 days, 11–17 days and > 17 days, and < 11 days and > 17 days, respectively. Those were compared using Tukey’s test after rank transformation and Fisher’s exact test with the Bonferroni method

Comparing different time interval groups, the group with a time interval of 11–17 days had a significantly lower proportion of stoma formation than the group with a time interval of > 17 days and fewer hospital days than the group with a time interval of < 11 days. The group with a time interval of < 11 days had a lower proportion of laparoscopic approach and a higher proportion of patients receiving adjuvant chemotherapy than the > 17 days group. There was no significant difference in operation time, estimated blood loss, harvested LNs, or postoperative complications among the time interval groups (Fig. 1).

### Long-term outcomes and risk factors

**Fig. 2:**
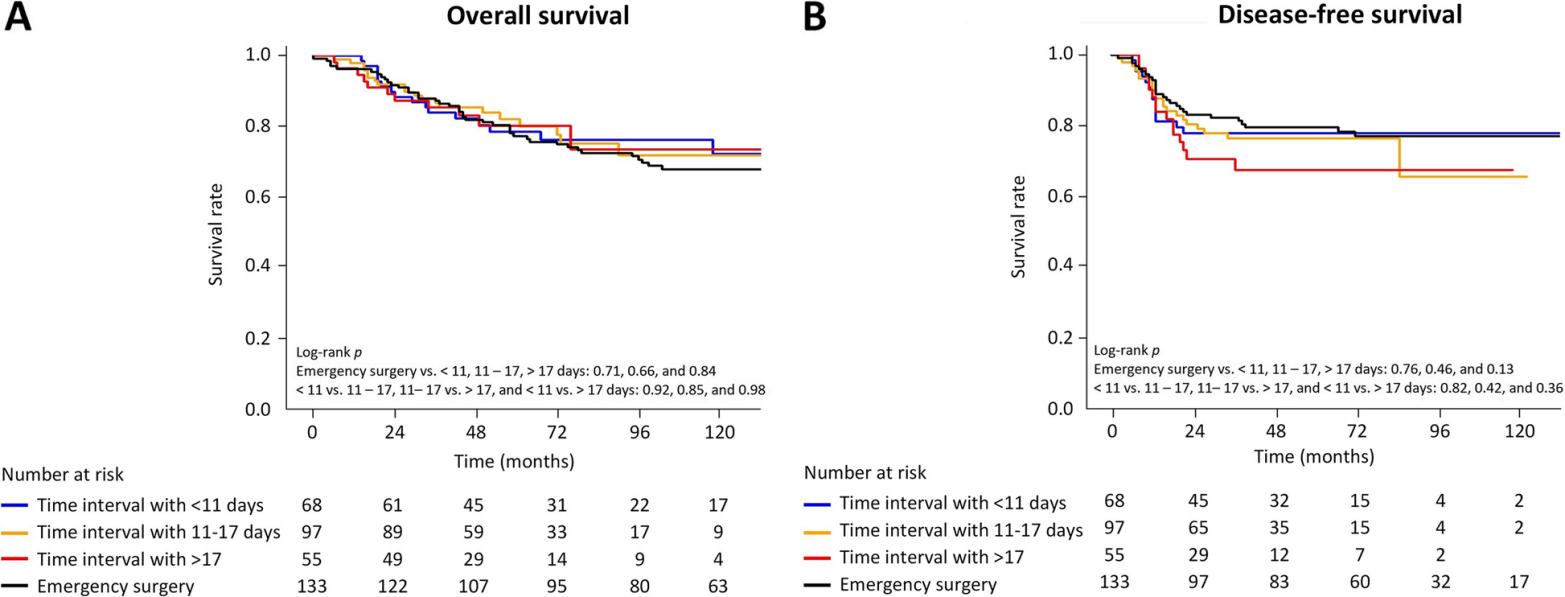
Comparison of overall survival (**A**) and disease-free survival (**B**) between self-expandable metal stent time interval groups and the emergency surgery group

Deaths were observed in 42 of 133 patients of the ES group, 16 of 68 patients in the SEMS group with a time interval of < 11 days, 20 of 97 patients in the SEMS group with a time interval of 11–17 days, and 11 of 55 patients in the SEMS group with a time interval of > 17 days during the median follow-up of 84 months [interquartile range (IQR): 48–116 months] (Supplementary Table 1). The OS did not differ significantly between the ES group and the SEMS group regardless of the time interval (< 11 days, 11–17 days, and > 17 days) (log-rank *p* = 0.71, 0.66, and 0.84, respectively) (Fig. 2). In multivariable Cox regression analysis, the group with a time interval of 11–17 days showed a lower risk of mortality than the ES group (hazard ratio (HR) = 0.48, 95% CI 0.4–0.97, *p* = 0.036). The OS of the groups with a time interval of < 11 days (HR = 0.82, 95% CI 0.38–1.75, *p* = 0.99) or > 17 days (HR = 0.44, 95% CI 0.17–1.10, *p* = 0.09) was not different from that of the ES group. Age (HR = 1.06, 95% CI 1.03–1.08, *p* < 0.001), preoperative CEA level (HR = 1.32, 95% CI 1.11–1.58, *p* = 0.002), and venous invasion (HR = 2.79, 95% CI 1.60–4.88, *p* < 0.001) were identified as independent factors associated with OS (Table 3).

**Table 3:** Multivariable analysis for risk of overall survival

Variables	Univariable	Multivariable		
	*P*	HR	95% CI	*P*
Primary treatment				
Emergency surgery^a^	0.95			0.034
Time interval of < 11 days	0.99	0.82	0.38–1.75	0.99
Time interval of 11–17 days	0.99	0.48	0.24–0.97	0.036
Time interval of > 17 days	0.99	0.44	0.17–1.10	0.09
Age	< 0.001	1.06	1.03–1.08	< 0.001
Sex				
Male^a^/female	0.13			
BMI	0.93			
ASA				
I–II^a^/III	< 0.001	1.93	0.97–3.82	0.06
Preoperative CEA	< 0.001	1.32	1.11–1.58	0.002
Location				
Right^a^	0.29			
Left	0.99			
Rectum	0.62			
Pathologic size	0.19			
Differentiation				
Undifferentiated	0.48			
Pathologic stage				
I–II^a^/III	0.06	0.90	0.52–1.58	0.72
Lymphatic invasion				
Present	0.011	1.56	0.95–2.55	0.08
Venous invasion				
Present	< 0.001	2.79	1.60–4.88	< 0.001
Peri-neural invasion				
Present	0.51			
Microsatellite instability test				
Low or stable^a^/high	0.20			
Adjuvant chemotherapy	< 0.001	0.74	0.44–1.23	0.24

^a^Reference. *ASA* American Society of Anesthesiologists, *BMI* body mass index, *CEA* carcinoembryonic antigen

Recurrences were observed in 27 of 133 patients of the ES group, 14 of 68 patients in the SEMS group with a time interval of < 11 days, 21 of 97 patients in the SEMS group with a time interval of 11–17 days, and 15 of 55 patients in the SEMS group with a time interval > 17 days during a median follow-up of 50 months (IQR 18–77 months) (Supplementary Table 1). There was no significant difference in DFS between the ES group and the time interval groups (< 11 days, 11–17 days, and > 17 days) (log-rank *p* = 0.76, 0.46, and 0.13, respectively) (Fig. 2). The recurrence risk did not differ between ES and the time interval groups (*p* = 0.88) (Table 4). Age (HR = 1.03, 95% CI 1.01–1.05, *p* = 0.013) and venous invasion (HR = 1.84, 95% CI 1.06–3.21, *p* = 0.031) were identified as risk factors for recurrence (Table 4).

**Table 4:** Multivariable analysis for risk of recurrence

Variables	Univariable	Multivariable		
	*P* value	HR	95% CI	*P* value
Primary treatment				
Emergency surgery^a^	0.53			0.88
Time interval of < 11 days	0.99	0.96	0.41–2.26	0.99
Time interval of 11–17 days	0.99	0.80	0.38–1.71	0.99
Time interval of > 17 days	0.43	1.02	0.43–2.44	0.99
Age	0.004	1.03	1.01–1.05	0.013
Sex				
Male^a^/female	0.11			
BMI	0.90			
ASA				
I–II^a^/III	0.038	1.79	0.90–3.57	0.10
Preoperative CEA	0.028	1.17	0.98–1.41	0.09
Location				
Right^a^	0.34			
Left	0.99			
Rectum	0.44			
Pathologic size	0.76			
Differentiation				
Undifferentiated	0.67			
Pathologic stage				
I–II^a^/III	< 0.001	1.76	0.99–3.12	0.05
Lymphatic invasion				
Present	0.011	1.12	0.67–1.86	0.66
Venous invasion				
Present	< 0.001	1.84	1.06–3.21	0.031
Peri-neural invasion				
Present	0.014	1.64	0.99–2.70	0.05
Microsatellite instability test				
Low or stable^a^/high	0.18			
Adjuvant chemotherapy	0.23			

^a^Reference. *ASA* American Society of Anesthesiologists, *BMI* body mass index, *CEA* carcinoembryonic antigen

**Fig. 3:**
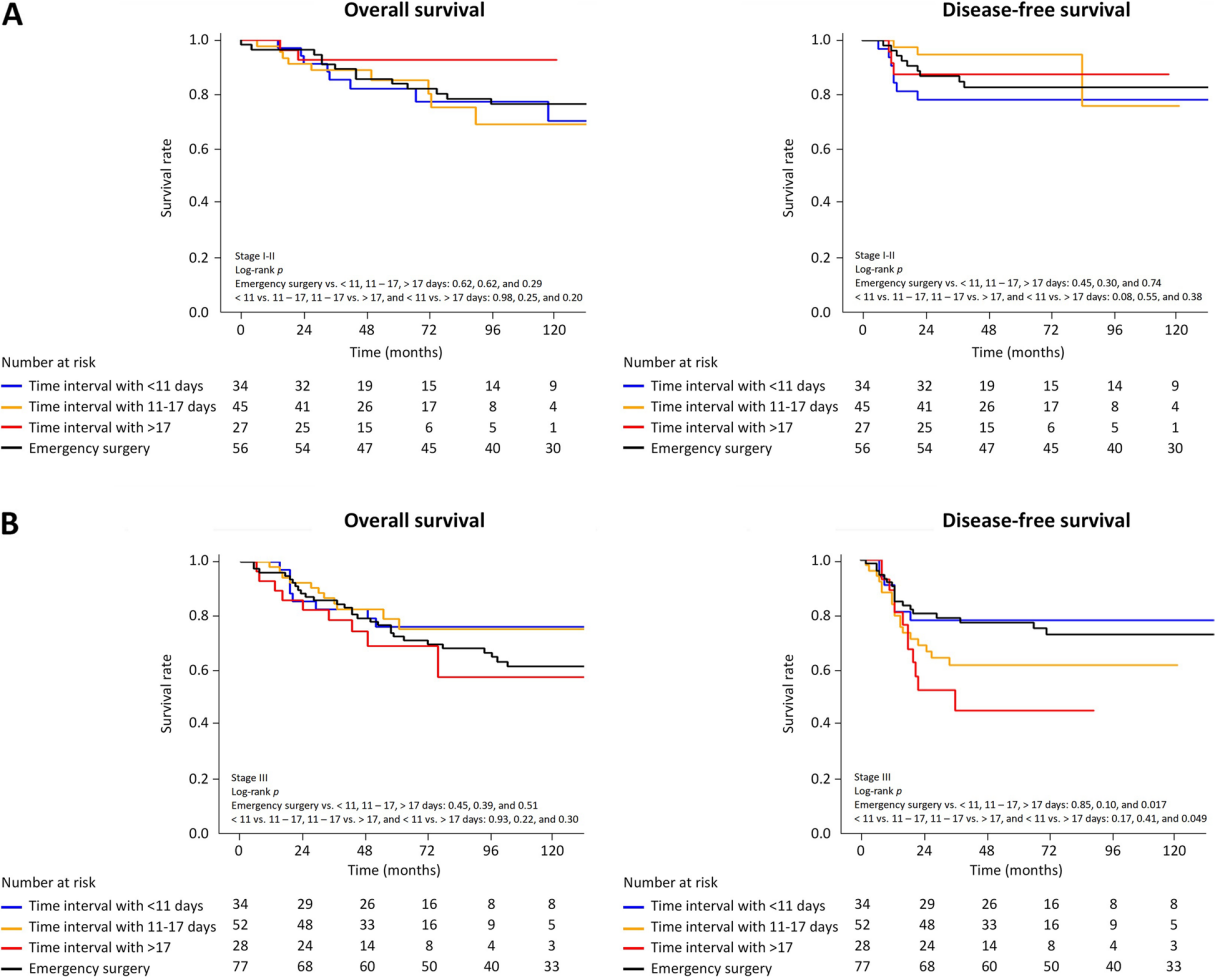
Comparison of overall survival and disease-free survival between self-expandable metal stent time interval groups and the emergency surgery group. **A** Patients with stage I or II disease. **B** Patients with stage III disease

A sub-analysis of stage I–II patients showed no significant difference in OS and DFS between ES and the time interval groups (Fig. 3). In the stage III subgroup, OS did not show significant differences between the ES group and the time interval groups of SEMS. However, DFS was significantly lower in the ES group (log-rank *p* = 0.017) and the time interval group of > 17 days (log-rank *p* = 0.049) than in the < 11 days group.

## Discussion

This study found that patients who received SEMS had better surgical outcomes, including a higher proportion of laparoscopic approaches, lower EBL, and shorter hospital stays after admission for surgery than those who received ES. The incidence of major adverse events after surgery was also lower in the SEMS group than in the ES group, although the difference was not statistically significant. The optimal time interval for surgery after stenting was found to be 11–17 days since patients in this group had shorter hospital stays and a lower rate of stoma formation compared to those in groups with time intervals of < 11 or > 17 days. The time interval group of < 11 days showed a lower proportion of laparoscopic approaches than those of > 17 days. Only the group with a time interval of 11–17 days exhibited a reduced risk of OS compared to the ES group in the multivariable analysis. However, in Kaplan–Meier analysis, no significant difference was observed in OS or DFS between time interval groups (< 11 days, 11–17 days, and > 17 days) and the ES group; in the subgroup analysis of stage III, the time interval group of > 17 days had a lower DFS than the ES group and the time interval group of < 11 days. Therefore, a time interval of 11–17 days was presumed to be optimal.

The strength of this study was that it was the first study to compare oncologic safety between SEMS time interval groups and an ES group. Despite clinical interest in appropriate time intervals from stenting to surgery, previous studies have yet to compare SEMS groups categorized by the time interval with an ES group. In addition, we included significantly more patients than in previous studies.

Our results support an optimal time interval of around 2 weeks, consistent with the updated ESGE guidelines [1] and a recent multicenter study [18]. In the multicenter study, a time interval of > 17 days showed a lower proportion of laparoscopic resections compared to a time interval of 5–10 days. Contrarily, postoperative complications were more frequent in the group with a time interval of 5–10 days than in the group with a time interval of > 17 days, whereas there was no significant difference in surgical outcomes between time intervals of 5–10 days and 11–17 days or between time intervals of 11–17 days and > 17 days [18]. In previous studies, a time interval of ≥ 10 days was associated with primary anastomosis [22], and a time interval of ≤ 15 days had a lower risk of postoperative complications than other intervals [23], whereas the risk of recurrence increased in those with a time interval of ≥ 18 days [24]. Furthermore, in a recent study, the DFS rate was significantly worse in those with a time interval of ≥ 16 days than in those with a time interval of < 16 days, with a time interval of ≥ 16 days reported as an independent risk factor for recurrence [25]. In contrast, a recent study suggested that a time interval of < 8 days had more favorable long-term outcomes, such as DFS and OS, than a time interval of 8–14 days or > 14 days [19]. However, the reason for such different findings was unclear due to the retrospective nature of the study. Based on accumulated reports, an interval of about 2 weeks to surgery is acceptable. Our results also support this finding.

It remains unclear why an optimal time interval is crucial and which factors contribute to such findings. A short break between stenting and elective surgery might be associated with insufficient restoration from ischemic injury or bowel edema. An ischemic injury could occur incidentally, ranging from 1 to 7% in patients with malignant bowel obstruction due to upstream bowel distension or abnormal stagnant intestinal bacteria [26–28]. Bowel edema changes within 1–2 weeks after stenting were also related to complex manipulation during surgery [29]. Given these factors, it is imperative to have an appropriate interval that allows for sufficient recovery from ischemic colitis associated with malignant obstruction and provides time for any bowel edema resulting from stenting to subside. It is also ambiguous why a longer break was associated with a poorer prognosis. Recent studies consistently showed that a longer interval was associated with a poorer prognosis. Especially, a time interval ≥ 35 days significantly increased the risk of recurrence (HR = 16.6, 95% CI 2.21–125) [19, 25]. Regarding potential factors, the pressure effect of SEMS placement might induce the dissemination of tumor cells. This is supported by previous studies showing increased perineural invasion in specimens after surgery [30, 31]. However, the impact of perineural invasion on long-term outcomes remains inconsistent [31–33]. Interestingly, our analysis found that the SEMS group had a higher proportion of perineural invasion than the ES group, although this did not affect OS or recurrence. Another concern is that stent insertion might increase the levels of viable circulating tumor cells [34], circulating CK20 mRNA [35], cell-free DNA, and circulating tumor DNA [36] in peripheral blood based on molecular studies. However, fewer than 30 patients were in the SEMS group in those studies, and some patients were in the palliative care stage. Furthermore, those studies could not verify adverse effects on long-term outcomes. Overall, a very long interval from SEMS placement to elective surgery seems to be related to an unfavorable prognosis.30, 31

This study had several limitations. There might be selection bias owing to its retrospective nature. All patients in the ES group were initially admitted to the emergency room and subsequently treated by ES. Some patients in the SEMS group initially visited outpatient clinics, not the emergency room. However, patients in the SEMS group were selected based on precise inclusion and exclusion criteria. We found that those in the time interval group of 11–17 days showed improved OS compared to those in the ES group in the multivariable analyses. Further studies are necessary to validate this finding and identify the weighted factors. We included patients with rectal cancer in this study, despite their low proportion. The approach to treating colon and rectal cancer is slightly different, such as neoadjuvant therapy. Thus, there might be the possibility of heterogeneity [37, 38].

In this study, those with a time interval of 11–17 days showed better surgical outcomes than those with time intervals of < 11 days or > 17 days. There was no significant difference in long-term effects between time intervals after stenting and ES. After adjusting for other factors, a potential OS benefit was found for a time interval of 11–17 days. Our study findings suggest that a time frame of approximately 2 weeks between stenting and elective surgery is beneficial for managing obstructive colorectal cancer patients.

### Supplementary Information

**Additional file 1. **Overall mortality and recurrence rate.

## Data Availability

Data underlying this article are available from the corresponding author upon reasonable request.
