# Supplementary material for: The optimal surgery timing after stenting in colorectal cancer patients with malignant obstruction: additionally compared with emergency surgery
Source: World J Surg Oncol. 2023 Aug 23;21:259. doi: 10.1186/s12957-023-03130-6 (PMC10463965; doi:10.1186/s12957-023-03130-6)
Supplement: Supplementary file 1 — Additional file 1. Overall mortality and recurrence rate. [file 12957_2023_3130_MOESM1_ESM.docx]

**Supplementary Table 1** Overall mortality and recurrence rate

|  | Emergency surgery  (*n =* 133) | SEMS (*n =* 220) | | |
| --- | --- | --- | --- | --- |
|  |  | Time interval <11 days  (*n =* 68) | Time interval 11-17 days  (*n =* 97) | Time interval >17 days  (*n =* 55) |
| Death (%) | 42 (31.6) | 16 (23.5) | 20 (20.6) | 11 (20.0) |
| Recurrence (%) | 27 (20.3) | 14 (20.6) | 21 (21.7) | 15 (27.3) |
